# Central nervous system diseases related to pathological microglial phagocytosis

**DOI:** 10.1111/cns.13619

**Published:** 2021-03-02

**Authors:** Ke Wang, Jiaying Li, Yue Zhang, Yichen Huang, Di Chen, Ziyu Shi, Amanda D. Smith, Wei Li, Yanqin Gao

**Affiliations:** ^1^ State Key Laboratory of Medical Neurobiology MOE Frontiers Center for Brain Science Institutes of Brain Science Fudan University Shanghai China; ^2^ Geriatric Research Education and Clinical Center Veterans Affairs Pittsburgh Health Care System Pittsburgh PA USA

**Keywords:** microglial phagocytosis, myelin, neuronal cell body, synapse

## Abstract

Microglia are important phagocytes of the central nervous system (CNS). They play an important role in protecting the CNS by clearing necrotic tissue and apoptotic cells in many CNS diseases. However, recent studies have found that microglia can phagocytose parts of neurons excessively, such as the neuronal cell body, synapse, or myelin sheaths, before or after the onset of CNS diseases, leading to aggravated injury and impaired tissue repair. Meanwhile, reduced phagocytosis of synapses and myelin results in abnormal circuit connections and inhibition of remyelination, respectively. Previous studies focused primarily on the positive effects of microglia phagocytosis, whereas only a few studies have focused on the negative effects. In this review, we use the term "pathological microglial phagocytosis" to refer to excessive or reduced phagocytosis by microglia that leads to structural or functional abnormalities in target cells and brain tissue. The classification of pathological microglial phagocytosis, the composition, and activation of related signaling pathways, as well as the process of pathological phagocytosis in various kinds of CNS diseases, are described in this review. We hypothesize that pathological microglial phagocytosis leads to aggravation of tissue damage and negative functional outcome. For example, excessive microglial phagocytosis of synapses can be observed in Alzheimer's disease and schizophrenia, leading to significant synapse loss and memory impairment. In Parkinson's disease, ischemic stroke, and traumatic brain injury, excessive microglial phagocytosis of neuronal cell bodies causes impaired gray matter recovery and sensory dysfunction. We therefore believe that more studies should focus on the mechanism of pathological microglial phagocytosis and activation to uncover potential targets of therapeutic intervention.

## Graphical abstract

Microglial excessive and insufficient phagocytosis of neuronal cell body, synapses, or myelin sheaths can aggravate CNS diseases.

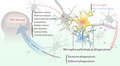

## 1 INTRODUCTION AND CLASSIFICATION OF PATHOLOGICAL MICROGLIAL PHAGOCYTOSIS

As the dominant phagocytes in the central nervous system (CNS), microglia play an important role in the removal of overproduced synapses,^1^ neurons,^2^ and myelin^3^ during neurogenesis, as well as in synapse pruning for neuronal modulation in the adult brain. However, under pathological conditions, the effect of microglial phagocytosis is considered to be paradoxical.^4^ On one hand, microglia phagocytosis may prevent secondary apoptosis‐induced inflammation by releasing anti‐inflammatory cytokines and by inhibiting pro‐inflammatory cytokines.^5^ On the other hand, microglial phagocytosis appears to activate respiratory burst, resulting in the release of neurotoxic reactive oxygen species (ROS).^4^ More importantly, in addition to phagocytosis of doomed‐to‐die cells, microglia also engulf viable cells and subsequently exacerbate brain injury or disturb normal development^6^ (eg, in the penumbra area of stroke^7^ or in neurodegenerative diseases^8^). Evidence suggests that this detrimental form of phagocytosis may be attributed to disease‐induced inflammation, subtoxic insult, or the release of attractant signals from neighboring engulfed dead cells that disable the ability of microglia to distinguish viable neurons from dying neurons.^6^, ^9^ Brown et al. have termed this kind of phagocytosis “phagoptosis,” which refers to excessive phagocytosis that leads to cell death.^8^ In addition to viable neurons, microglia have been reported to adhere to and phagocytose intact myelin fibers, rather than myelin debris, thereby exacerbating white matter injury in a chronic cerebral hypoperfusion mouse model.^10^ Insufficient phagocytosis contributes to pathology as well. For example, failure to prune synapses during development may account for autism‐like behavior in adult brains.^11^ In addition, insufficient clearance of myelin debris possibly explains why axonal regeneration cannot occur^4^ after spinal cord injury.

Under pathological conditions, microglial phagocytosis can have both beneficial and detrimental effects on the recovery of the CNS to injury or disease. In the current review, we focus on the detrimental effects of microglial phagocytosis that occur under pathological conditions, which lead to structural or functional abnormalities of surviving cells and tissue. These negative effects of microglial phagocytosis can be driven by unwarranted or misdirected phagocytosis, which we deem to be excessive phagocytosis, or reduced phagocytosis. Thus, pathological microglial phagocytosis may not only serve as a cause of neurological disease but may also exacerbate or facilitate progression of the disease. In contrast to normal warranted microglial phagocytosis, pathological microglial phagocytosis may include engulfment of viable cells that should not be phagocyted. Pathological microglial phagocytosis can be divided into two broad categories based on the degree of the phagocytic activity of microglia: excessive phagocytosis and reduced phagocytosis. Within these two categories, pathological microglial phagocytosis can be further subcategorized based on whether the target is synapses, neuronal cell bodies, or myelin. Excessive phagocytosis by microglia of synapses, neuronal cell bodies, or myelin sheath can be detrimental to tissue and functional recovery if unwarranted or misdirected and can exacerbate CNS diseases. On the other hand, reduced microglial phagocytosis, such as reduced phagocytosis of synapses or myelin debris, may lead to pathological connectivity and functional disorders, respectively. Examples of these subtypes of pathological microglial phagocytosis and related signaling will be discussed in turn below.

## 2 SIGNAL PATHWAY OF PATHOLOGICAL MICROGLIAL PHAGOCYTOSIS

Phagocytosis is such a rapid process that cell debris or dead cells are rarely detected under normal physiological conditions. Phagocytosis can be divided into four steps. The first step requires chemotaxis termed “find‐me” signals released by apoptotic or stressed cells to announce their existence and to recruit phagocytes. Second, microglia recognize the exposed “eat‐me” signals on the surface of the apoptotic or compromised cells. Additionally, the presence of “don't eat‐me” signals enables microglia to distinguish living cells from dead targets. The Ras homologous (RHO) protein family is often involved in this process. The third step is internalization or engulfment of the targets, involving the remodeling of microtubule proteins in the cytoplasm of phagocytes, often through orchestration of the RHO protein family. Following internalization, phagosomes that contain corpse contents become increasingly acidic and eventually fuse with lysosomes that contain the digestive enzymes essential for degradation. Whether phagocytosis is beneficial or detrimental, the intracellular mechanism is similar. Hence, what truly determines the fate of dead or living targets is the exposure of eat‐me signals or don't eat‐me signals. Therefore, inhibition of the inappropriate exposure of these signals may prevent the death of innocent cells and promote the execution of detrimental targets, thereby mitigating diseases.

### 2.1 “Eat Me” SIGNALING PATHWAY

#### 2.1.1 Components of the “eat‐me” signaling pathway

“Eat‐me” signals can be divided into two categories—membrane‐anchored signals and soluble bridging molecules.^12^ In the nervous system, phosphatidylserine (PtdSer) is the most common membrane‐anchored “eat me” signal. Normally confined to the inner leaflet of the plasma membrane, PtdSer itself is not toxic to neurons; however, exposure of PtdSer at the early onset of apoptosis marks the neuron for selective engulfment.^8^ Notably, PtdSer is reversibly exposed in viable but stressed neurons, providing for the possibility for re‐internalization of PtdSer and rescue of these neurons.^13^ The receptors that bind directly to PtdSer include members of the T cell/transmembrane, immunoglobulin, and mucin (TIM) family (TIM1, 3, 4), the seven transmembrane brain angiogenesis inhibitor 1 (BAI1) receptor, and Stabilin 2 (atypical EGF‐motif containing membrane protein). Calreticulin is another important membrane‐anchored “eat‐me” signal and is normally localized in the endoplasmic reticulum.^14^ Exposed calreticulin induces phagocytosis by binding to the low‐density lipoprotein receptor‐related protein (LRP) that is located in microglia.^15^ Unlike membrane‐anchored eat‐me signals, which directly bind to receptors on the microglial surface, soluble bridging molecules with at least two binding domains serve as a link between the membrane‐anchored signal and phagocytic receptors. For example, milk fat globule epidermal growth factor 8 (MFG‐E8), which is released from microglia and astrocytes during inflammation, can bind to exposed PtdSer and the vitronectin receptor (VNR). Inflammatory cytokines mediate the upregulation of Mer tyrosine kinase (MerTK), which also acts as a microglial phagocytic receptor that mediates phagocytosis of apoptotic cells, stressed neurons, and synapses. Notably, MerTK interacts with PtdSer through two soluble bridging molecules, Gas6 and Protein S, whose N‐terminal 11 γ‐carboxyglutamic acid residues can bind to PtdSer.^12^ Microglia also release annexin 1, which binds to neuronal PtdSer and activates microglial formyl peptide receptor 2.^16^ In addition, the complement components, C1q and C3, which are released by microglia and astrocytes, also act as soluble bridging eat‐me signals. C1q can bind to neuronal surface de‐sialylated glycoprotein. It can be recognized by LRP and promote C3 conversion to C3b. The latter is detected by CR3, thereby promoting microglial phagocytosis of tagged amyloid, synapses, or neurons.^8^, ^17^ Furthermore, neurons and oligodendrocytes are particularly vulnerable to complement‐mediated death due to the lack of surface complement regulatory protein decay activating factor.^18^ Triggering receptor expressed on myeloid cells 2 (TREM2) is another phagocytosis receptor.^19^ Its corresponding “eat me” signal, TREM2‐L, is found on Neuro2A cells, cultured cortical, and dopamine neurons.^20^ Of note, it has not been yet established whether other molecules released by apoptotic or stressed cells also play an “eat‐me” role in the phagocytosis process.

#### 2.1.2 Abnormal activation of the “eat‐me” signaling pathway

**FIGURE 1:**
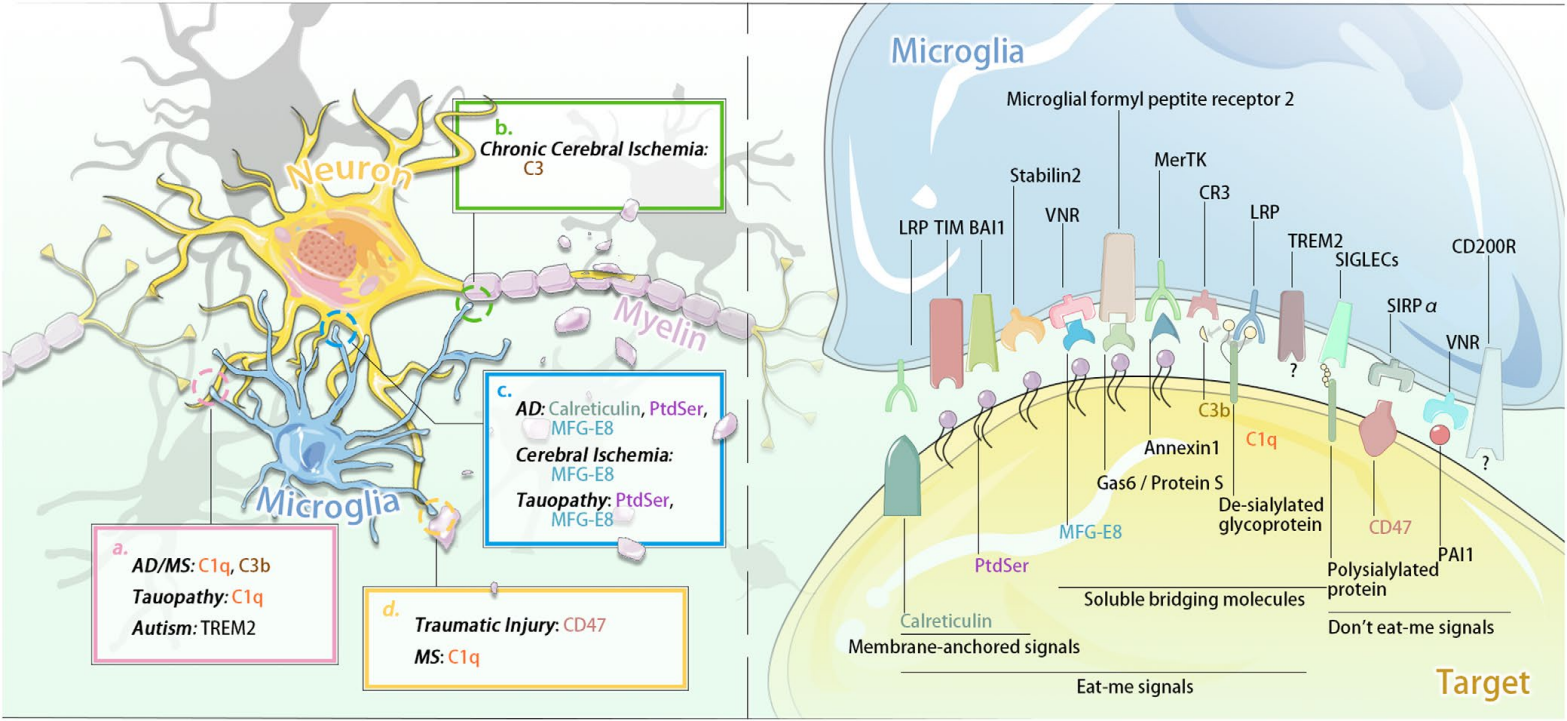
Signals and molecules of pathological phagocytosis between microglia and target cell. Left panel: (A) Pathological microglial phagocytosis of synapse. (B) Pathological microglial phagocytosis of myelin sheath. (C) Pathological microglial phagocytosis of neuronal cell body. (D) Pathological microglial phagocytosis of myelin debris. The abnormal activation of "eat‐me" and "don't eat‐me" signaling pathways exists in many CNS diseases. For example, Aβ causes C1q's activation and induces excessive microglial phagocytosis of synapses (pink a). In chronic cerebral ischemia, excessive microglial phagocytosis of myelin sheaths via C3/C3aR pathway (green b). Right panel: In CNS disease, microglia recognize the "eat‐me" and "don't eat‐me" signals exposed on the surface of target cells. "Eat‐me" signals can be divided into two categories: membrane anchoring signals (eg, TIM, PtdSer) and soluble bridging molecules (eg, CR3, C1q). "Don't eat‐me" signal mediates the inhibition of microglial phagocytosis, which is mainly composed of CD47‐SIRPα

Abnormal activation of the “eat‐me” signaling pathway occurs in many diseases (Figure 1, right panel). While accumulation of high concentrations of amyloid beta (Aβ), a leading cause of AD, can induce direct toxicity to neurons, low concentrations can also elicit neuronal loss owing to microglial phagocytosis. Evidence suggests that Aβ greatly increases the phagocytic capacity of microglia and promote neuronal PtdSer exposure, which induces phagocytosis.^15^ Other studies have found that MFG‐E8 and calreticulin also contribute to phagocytosis. Either blocking calreticulin or PtdSer exposure, or knocking out neuronal MFG‐E8 can prevent AD‐induced neuronal loss,^21^ indicating that phagocytosis can directly result in neuronal death and might therefore contribute to neurodegeneration. As occurs with Aβ accumulation, tau aggregation, which mediates ROS production, also leads to neuronal PtdSer exposure with subsequent promotion of nitric oxide (NO) production in the phagocytes, resulting in increased release of MFG‐E8 and excessive phagocytosis of live neurons.^22^ Loss‐of‐function mutation of TREM2 is sufficient to induce Nasu‐Hakola disease, a neurodegenerative disorder in which microglial capacity to phagocytose membrane debris is impaired, contributing to subsequent inflammation and cell death.^23^

In the peri‐infarct region of the cerebral ischemic area, the neurons are stressed but still alive. Under this condition, PtdSer exposure triggers microglial phagocytosis that leads to the direct death of the neurons. Preventing phagocytosis of these compromised neurons by knocking out MerTK or MFG‐E8 can restore these stressed and viable neurons, thereby improving outcomes of ischemia.^7^ After chronic cerebral hypoperfusion, microglia aggregate around the intact myelin fibers and phagocytose myelin components via the C3/C3aR pathway. Ablation of C3aR can inhibit the aberrant phagocytosis and restore white matter integrity.^10^ Parkinson's disease (PD) results from progressive neuronal loss of neuromelanin containing neurons in the substantia nigra. Neuromelanin, released from dying cells and recognized by microglia, induce inflammation and subsequent neuronal loss. CR3 deletion can prevent neuronal loss, suggesting the possibility that in the context of PD pathology, microglial phagocytosis may be involved in PD progression.^6^ The above findings only demonstrate that these molecules play a regulatory role in microglial phagocytosis under pathological conditions. However, it does not mean that alteration of these signaling molecules are exclusively involved in pathological phagocytosis, and it is not yet known whether any of the above signaling pathways can distinguish microglial pathological phagocytosis from normal microglial phagocytosis.

### 2.2 “Don't eat‐me” signaling pathway

#### 2.2.1 Component of the “don't eat‐me” signaling pathway

In contrast to “eat‐me” signals, “don't eat‐me” signals inhibit microglial phagocytosis. CD47, an important “don't eat‐me” signaling molecule, well characterized in the immune system, is expressed on myelin, myeloid cells, red blood cells, platelets, neurons, fibroblasts, and endothelial cells.^2^ CD47 directly inhibits aberrant phagocytosis by binding to receptor signal regulatory protein‐α (SIRPα), which is expressed on phagocytes and neurons. Previous studies have indicated that CD47 may localize to synapses.^24^ In the developing retinogeniculate system, CD47‐SIRPα protect synapses from aberrant removal, while CD47‐deficient microglia in turn fails to display phagocytotic preference to less active input.^25^ In addition, polysialylated proteins on neurons also inhibit phagocytosis by binding to receptors on microglia, and by activating sialic acid‐binding immunoglobulin‐like lectins (SIGLECs), such as SIGLEC‐11 (in humans) and SIGLEC‐E (in mice).^8^

Other molecules that may mediate the “don't eat‐me” signaling pathway are CD200, plasminogen activator inhibitor 1 (PAI1), and CD24. CD200 is broadly expressed on neurons. The receptor of CD200, CD200R, has been reported to exert inhibitory effects on microglial phagocytosis^19^, ^26^; however, whether CD200 truly acts as a “don't eat‐me” signal in the nervous system remains unknown. PAI1, which is released by microglia and astrocytes, can inhibit VNR‐mediated phagocytosis,^8^ though it induces microglial migration to prey cells. CD24 has recently been uncovered as a novel putative “don't eat‐me” signal, which is upregulated in the tumor compartment and helps tumors escape phagocytosis by binding tumor‐associated macrophages to SIGLEC‐10 on the surface of macrophages.^27^ Although the phagocytosis‐related role of CD24 in the nervous system is not yet fully established, upregulation of CD24 is found in TBI^28^ and in experimental autoimmune encephalomyelitis (EAE) (a model for MS), as well as transiently, on developing neurons,^29^ which may suggest a potential anti‐phagocytic role of CD24 in nervous system disease.

#### 2.2.2 Abnormal activation of the “don't eat‐me” signaling pathway

Limited studies so far have focused on the role of “don't eat‐me” signals in nervous system development or diseases (Figure 1, right panel). Hutter et al. demonstrated that disruption of CD47‐SIRPα reduced glioblastoma multiforme (GBM) by inducing tumor clearance by tumor‐associated macrophages and microglia.^30^ Although CD47‐SIRPα interaction is considered beneficial because it inhibits phagocytosis of intact myelin, the same mechanism also underlies poor axonal regeneration after traumatic injury to the peripheral nervous system (PNS) or CNS due to insufficient clearance of degenerated myelin.^2^

Although some studies have shown that upregulation of CD47‐SIRPα decreases the phagocytic activity of microglia,^2^, ^25^, ^30^ there is no direct evidence that the upregulation of CD47‐SIRPα inhibits normal microglial phagocytosis or excessive microglial phagocytosis under pathological conditions. Thus, further studies are needed to determine if there is truly a causal link between CD47‐SIRPα and the phagocytic activity of microglia. Meanwhile, known constituent molecules of “eat‐me”/“don't eat‐me” signaling pathways (Figure 1, right panel) and their abnormal activation in different nervous system diseases (Figure 1, left panel) are summarized in Figure 1.

## 3 NERVOUS SYSTEM DISEASE RELATED TO PATHOLOGICAL MICROGLIAL PHAGOCYTOSIS

**FIGURE 2:**
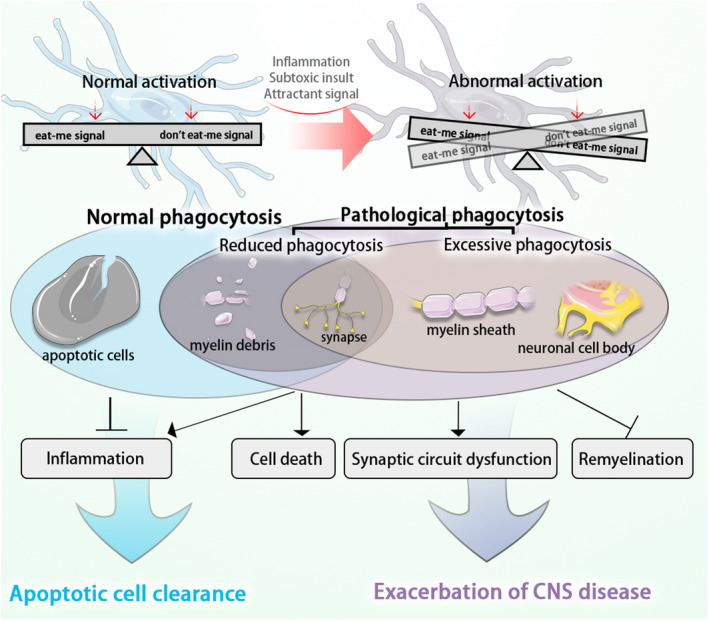
The comparison between normal phagocytosis and pathological phagocytosis. In CNS diseases, microglia phagocyte apoptotic cells, myelin debris, and damaged synapses. They play a beneficial role in neuroinflammation resolution and removal of apoptotic cells. This kind of phagocytosis can be considered normal phagocytosis. However, pathological microglial phagocytosis may occur as a result of abnormal activation of microglia. Pathological microglial phagocytosis includes reduced phagocytosis of myelin debris or synapses, and/or excessive phagocytosis of synapses, myelin sheath, and neuronal cell bodies. Reduced and excessive phagocytosis contributes to cell death, synaptic circuit dysfunction, and demyelination, ultimately resulting in exacerbation of CNS diseases

Microglia can phagocytose cell debris and overproduced synapses. Beneficial phagocytosis, which we refer to “normal microglial phagocytosis”, facilitates structural and functional recovery of impaired tissue. Alternatively, microglial phagocytosis can also be harmful. This kind of phagocytosis, which we term “pathological microglial phagocytosis,” includes unwarranted, or misdirected phagocytosis that disrupts tissue integrity. It also includes reduced phagocytosis, whereby detrimental and yet‐to‐be‐eaten substance debris is not effectively cleared from the diseased environment (Figure 2).

### 3.1 Pathological microglial phagocytosis of synapse

#### 3.1.1 Excessive phagocytosis of synapse

##### Alzheimer's disease (AD)

AD is a neurodegenerative disease of the CNS characterized by the pathological accumulation of plaques containing Aβ, phosphorylated tau (p‐Tau) protein, and synapse debris.^31^ It is recognized by the World Health Organization as a global public health priority, as around 44 million people around the world are currently suffering from dementia, with a greater than three‐time predicted increase by 2050 as the population ages.^32^ In addition to the aforementioned plaque accumulation, neurofilament aggregation, axonal dystrophy, enhanced astrocytes, and microglia activation, as well as amyloid angiopathy of the brain may also occur.^33^, ^34^, ^35^ Thus, due to enhanced microglia activation in AD, excessive phagocytosis may occur.

Many studies suggest that microglia play a biphasic role in AD. It has been found that activated microglia and Aβ co‐localize.^36^ On the one hand, microglia promote the decomposition of Aβ, which should be beneficial. On the other hand, microglia can be activated through complement systems to overly engulf synapses, which leads to a greater loss of synapses as AD progresses. The microglia cell surface receptor, TREM2, can trigger protein tyrosine phosphorylation. TREM2 can detect damage related lipid patterns during neurodegeneration in diseases such as AD, and maintain the response of microglia to Aβ accumulation.^37^ TREM2 stimulates the activation of signal transduction pathways that promote the chemotaxis, phagocytosis, survival, and proliferation of microglia.^38^ Complement systems mediate the phagocytosis of synapses by microglia. The complement component C1q is highly expressed in the adult brain.^39^ It has been shown that the combination of C1q and Aβ can trigger the activation of the classical complement cascade.^40^ Indeed, in an animal model of familial AD, early deposit of Aβ induces neuronal expression of C1q and C3, ultimately leading to excessive microglial phagocytosis of viable synapses.^1^, ^41^ Inhibition of C1q with antibodies, or genetically knocking out C3 effectively prevents synapse loss and functional deficits caused by Aβ deposit.^42^ A study in the amyloid precursor protein/presenilin 1 (APP/PS1) mouse model showed that the accumulation of C1q in APP/PS1 mice was increased in synapses containing large amounts of septin‐3 and septin‐5. A further study in mice and rats showed that blocking the metabotropic glutamate receptor 1 (mGluR1) inhibited the dephosphorylation of fragile X mental retardation protein (FMRP) and the local translation of synaptic C1q mRNA, with subsequent reduction in the pathological phagocytosis of synapse by microglia, correlated with restored synaptic and cognitive functions.^44^

##### Tauopathy

Microglia also secrete a large number of pro‐inflammatory factors and play an important role in tau pathology. In tauopathy, tau aggregates may lead to increased externalization of PtdSer on the surface of intact dendritic spines. One of the “eat‐me” signals, C1q can detect PtdSer exposure and subsequently bind to PtdSer, resulting in the removal of synapses. Benetatos et al. have validated that inhibition of the upstream molecule of PtdSer exposure, phosphatase and tensin homolog (PTEN), can prevent microglia‐mediated synapse loss.^45^

##### Schizophrenia

Excessive microglial phagocytosis also leads to synapse loss in schizophrenia. Schizophrenia is a common disease in young people in whom excessive phagocytosis of synapses culminates into a syndrome characterized by dysregulation of emotions and cognition, and the disharmony of mental activities. The main histopathological changes in schizophrenia are the loss of gray matter in prefrontal, temporal, and subcortical structures in the acute phase,^46^ the damage to white matter tracts connecting these areas, and the loss of synapses.^47^, ^48^ It was found that the number of activated microglia was increased in 15% of schizophrenic patients, and the morphology of microglia in the frontal cortex was altered.^49^ A study found that microglia in cortical tissue of schizophrenic patients pruned synapses excessively, resulting in decreased synaptic density and abnormal synaptic structure. Minocycline was found to reduce microglia‐mediated synaptic pruning in vitro and has since become an adjunct therapeutic treatment for schizophrenia. Also, schizophrenia risk‐related variants at the human complement component 4 site are associated with increased neuronal complement deposition and synaptic uptake. In schizophrenic patients’ induced pluripotent stem cell‐derived neural cell culture, C3 complement deposition was significantly positively correlated with the copy number of C4AL (long form of C4A), but not with the short or long copy number of C4B13, which indicates C3 is also involved in excessive microglial phagocytosis of synapses in schizophrenia.^50^

##### Rett Syndrome (RTT)

Excessive microglial phagocytosis of synapses is also considered to be the cause of Rett syndrome (RTT). RTT has a high incidence in girls and is characterized by autism‐like behavior, decreased motor control, irregular breathing, and neurodevelopmental disorders. Studies have shown that a methyl CpG binding protein 2 (MeCP2) mutation is the basis of RTT. Studies in mouse models have shown that both neurons and glia, especially microglia, are associated with this disease.^51^, ^52^ However, synaptic circuit dysfunction usually occurs before the pathological phenotype. The loss of MeCP2 in the RTT model of developing and adult mice led to contraction of mature dendrites of pyramidal neurons and significantly decreased the density of dendritic spines.^53^ Thus, excessive microglial phagocytosis may not only serve as the cause of RTT, but may also exacerbate synapse loss at late stage of disease^54^ as some studies suggest. Nevertheless, no relevant signaling pathway has been explored, and thus, further study of the role of microglia in the development of RTT is warranted.

##### Nasu‐Hakola Disease

TREM2 deficiency results in insufficient clearance of overproduced synapses during early brain development, accounting for some developmental disorders such as autism or Nasu‐Hakola disease, in which increased synaptic density and increased excitability of neurotransmission can be observed. Knocking out TREM2, which mimics Nasu‐Hakola diseases, leads to poor neural connection between the prefrontal and hippocampal regions and poor social behavior outcomes.^55^

##### Multiple Sclerosis (MS)

Excessive microglial phagocytosis of synapses may be the cause of MS. About three million people in the world are affected by MS, and the patients have motor, cognitive, and visual deficits in the course of the disease.^56^ The main pathological changes are extensive demyelination of white matter, destruction of the blood‐brain barrier, proliferation of glial cells, neuroinflammation, and synaptic loss.^57^, ^58^, ^59^, ^60^ Examination of MS patients and experimental autoimmune encephalomyelitis (EAE) mice showed that the number of synapses in the hippocampus, insula, frontal temporal occipital cortex, and striatum was significantly decreased.^61^, ^62^ Complement deposition combined with microglia phagocytosis has proven to be the mechanism underlying synaptic pruning in MS. The protein and mRNA levels of C1q and C3 increased in EAE mice. The loss of the C3 gene protected mice from EAE induced synaptic loss, reduced the activation of microglia, and enhanced the EAE clinical score.^63^ Studies on postmortem human MS patients, primates, and two rodent demyelinating disease models, show that synaptic loss is not associated with local demyelination and neuronal degeneration. The use of complement inhibitor CR2‐Crry on C3‐binding synapses reduced the excessive phagocytosis of synapses.^64^

#### 3.1.2 Reduced phagocytosis of synapse

##### Autism Spectrum Disorder (ASD)

The main pathological mechanism underlying ASD is distinct from other CNS diseases with regard to microglial phagocytosis in that there is a reduction in microglia phagocytosis of synapses as oppose to excessive phagocytosis, leading to abnormal synaptic circuit connections. ASD is a developmental disorder characterized by abnormal social skills, communication skills, interest, and behavior patterns.^65^, ^66^ Epidemiological survey results in recent years show that the incidence rate of ASD is about 0.3% and the incidence rate is increasing year by year.^67^ ASD affects boys about four times more than girls. In ASD, the proliferation of glial cells is significantly higher than normal. Positron emission tomography (PET) functional imaging scans showed that activation of microglia could be observed in the cerebellum, midbrain, pons, fusiform gyri, and anterior cingulate.^68^, ^69^ It is speculated that the damage to the phagocytic capacity of microglia will affect the normal pruning and maturation of developing synapses.^70^ For example, CX3CR1 knockout mice and TSC2^+^/mice exhibit deficits in synaptic pruning, impaired social behavior, and functional connectivity, all of which are characteristic of ASD.^71^ Constitutive activation of mammalian target of rapamycin (mTOR), resulting in decreased autophagy, is thought to underlie these deficits in TSC2^+^ mice as rapamycin, an mTOR inhibitor, decreased the symptoms and spinal pruning defects in these mice.^72^ Thus, modulation of mTOR activity may be an important target to increase phagocytosis of synapses by microglia in autism. Deficits in synaptic pruning could also be reversed by exercise in offspring subjected to maternal immune activation in utero.^73^ Voluntary wheel running was found to increase microglia phagocytosis of synapses. However, it remains unknown whether mTOR regulates total microglial phagocytic activity or only rescues phagocytic activity in microglia in which activity was reduced. Future studies are therefore required in order to establish whether mTOR is a specific target for modulating microglial phagocytosis under pathological conditions.

### 3.2 Pathological microglial phagocytosis of neuronal cell bodies

#### 3.2.1 Excessive phagocytosis of neuronal cell bodies

Previous studies have demonstrated that microglia can phagocyte live neurons.^8^, ^74^, ^75^, ^76^ Time‐lapse video was performed, and video imaging of cells in culture revealed that inflammation triggers microglial phagocytosis of neurons that appear to be healthy with no nuclear condensation discerned by light microscopy when engulfed (ie, neurons were healthy and viable without any characteristics of apoptosis or necrosis). Blocking microglia phagocytosis protected live neurons from death.^77^ In the following sections, we will discuss excessive microglial phagocytosis of live neurons in various nervous system diseases.

##### Parkinson's Disease (PD)

Excessive microglial phagocytosis of neuronal cell bodies can be found in PD. PD is the second most common neurodegenerative disease. It is more common in the elderly than in the young. PD in most patients is sporadic, as less than 10% of the patients have a family history. The most important pathological change in PD is the degeneration and death of dopaminergic neurons in the substantia nigra of the midbrain, which leads to the significant decrease in dopamine (DA) content in the striatum.^78^, ^79^ Excessive microglial phagocytosis of dopaminergic neurons in the substantia nigra may be an important pathogenic mechanism in the pathological process of PD. The pivotal molecule alpha‐synuclein (α‐syn) regulates microglia activation.^80^ α‐Syn‐induced microglia activation promoted phagocytosis via the microglial FcγR receptor, which led to exacerbated loss of DA neurons and other neurodegenerative changes in PD.^81^, ^82^ α‐Syn acts on other microglial receptors as well, such as the prostaglandin EP2 receptor, which regulates microglia activation and α‐syn aggregate phagocytosis.^83^ Purinergic P2X7 receptors also regulate α‐syn mediated microglial activation via nicotinamide adenine dinucleotide phosphate oxidase (PHOX),^84^ an enzyme that plays a pivotal role in superoxide production. In PD, increased expression of TREM2 and mannose receptor C‐type 1 (Mrc1) in microglia also promotes activation of phagocytosis,^38^, ^85^ which when excessive or unwarranted, as discussed previously, contributes to neurodegeneration. Further, the loss of Tyro3, Axl, and MerTK (TAM) phagocytic receptors, increased the survival time of mice overexpressing α‐syn A53 T, suggesting that excessive microglial phagocytosis of neuronal cell bodies by TAM receptors promotes neurodegeneration and leads to animal death.^86^

Rotenone, a pesticide that has been epidemiologically linked to PD,^87^ induces microglia activation and dopaminergic neuron loss. Rotenone increased the proliferation of microglia and phagocytosis of living neurons. Inhibition of the PY2 purinoceptor 6 (P2Y6) receptor or use of an anti PtdSer antibody inhibited this excessive phagocytosis and prevented the loss of living neurons.^88^ A study on the temporal relationship between microglia activation and neuronal loss showed that the activation of microglia preceded the loss of DA neurons, and that the degenerating neurons may have been engulfed by phagocytic microglia.^89^ A study has also found that LRRK2 can enhance the phagocytic capacity of microglia through the specific regulation of actin cytoskeleton regulatory factor WAVE2, followed by subsequent death of dopaminergic neurons,^90^ further suggesting that unwarranted or premature clearance of compromised DA neurons may facilitate the progression of PD, and thwart the action of endogenous repair mechanisms.

##### Ischemic Stroke (IS)

Ischemic stroke has a high incidence rate, high disability rate, and a high mortality. During the acute stage of ischemic stroke, microglia are activated in large quantities and phagocyte necrotic tissue, thus preventing the release of inflammatory and toxic substances that could aggravate damage.^91^, ^92^, ^93^, ^94^ One of the important roles of phagocytosis is to engulf injured neurons.^95^ However, in addition to apoptotic neurons, microglia can also engulf viable neurons. A study on the mechanism of signal transducer and activator of transcription 6/arginase 1 (STAT6/Arg1) in ischemic stroke found that microglia not only phagocyte a large number of apoptotic neurons, but also a small number of viable neurons after ischemic stroke. Knocking out STAT6 in mice not only inhibited microglia from phagocytizing apoptotic neurons, but also decreased their ability to phagocytize viable neurons.^96^ Some studies have shown that the dying neurons in the lesion area release chemokines such as CX3CL1, which cause “eat me” signals to be exposed, and subsequently recognized by C3aR‐expressing microglia, leading to the phagocytosis of living neurons. The C3aR antagonist SB290157 can reduce neuronal death by limiting secondary phagocytosis after stroke.^97^ Correspondingly, in our study (not yet published), we observed that a large number of activated microglia aggregated and phagocyted neurons in the peri‐infarct area in the acute phase of ischemic stroke. However, although the majority of the phagocyted damaged neurons (about 85%) are apoptotic neurons (Caspase3^+^NeuN^+^), a few neurons (about 15%) were still alive (Caspase3^−^NeuN^+^). Limited by the current technology, we are still unable to elucidate the differences between pathological microglial phagocytosis and normal microglial phagocytosis in ischemic stroke.

##### Tauopathy

Tau aggregates within living neurons, as characterized in many neurodegenerative diseases, contributes to externalization of PtdSer due to the production of ROS.^8^ Meanwhile, these neurons also stimulate microglia to secrete MFG‐E8 and NO, which together with PtdSer, orchestrate phagocytosis of living neurons. Preventing MFG‐E8 or NO production can rescue these living neurons with tau inclusions.^22^

##### Traumatic Brain Injury (TBI)

TBI is a head injury caused by an external force. The function of microglia in TBI is similar to that observed in ischemic stroke.^98^, ^99^ At different stages after TBI, the phagocytic phenotype of microglia changes, subsequently altering their function. A study of interleukin‐13 (IL‐13) in TBI showed that IL‐13 treatment improved the ability of microglia to phagocyte fluorescent latex beads or dead neurons. Although most of the neurons were apoptotic, several viable neurons were also phagocyted by microglia activated in the acute phase.^100^

### 3.3 Pathological microglial phagocytosis of myelin

#### 3.3.1 Excessive phagocytosis of myelin sheath

##### Ischemic Stroke (IS)

Normal phagocytosis of myelin debris by microglia is beneficial to the regeneration and repair of myelin, while excessive phagocytosis of myelin sheath exacerbates demyelination. In the bilateral common carotid artery occlusion/bilateral common carotid artery stenosis (BCCAO/BCAS) model of chronic cerebral ischemia, white matter damage is extensive. In addition, the number of oligodendrocytes was significantly decreased after chronic cerebral ischemia. The damage to white matter is associated with pathological microglial activation, adhesion, and excessive phagocytosis of myelin after stroke. Indeed, 14 days after stroke, a large number of microglia was observed surrounding, contacting, and excessively engulfing myelin sheath, which in turn caused myelin sheath damage.^10^ Twenty‐eight days after stroke, demyelination and massive exposure of non‐phosphorylated neurofilaments, as well as an increased SMI32/myelin basic protein (MBP) immunofluorescence ratio were observed.

#### 3.3.2 Reduced phagocytosis of myelin debris

##### Aging

As myelin debris inhibits remyelination, it needs to be promptly cleared by microglia. In the aging CNS, the reduced number, monitoring capacity, and phagocytic activity of aging microglia leads to a decreased ability of microglia to clear myelin debris, which in turn leads to slower remyelination compared with the young CNS.^101^ In a study of simulated demyelination using a lysophospholipid demyelinating toxin injection, it was observed that middle‐aged mice recruited fewer microglia than young mice 7 days after demyelination and did not reach the same level as young mice until 21 days post‐injection. Moreover, histological staining with Oil Red O indicated that phagocytosis of lipids in middle‐aged mice was lower than that of young mice 7 days after demyelination.^102^

(The main characteristics of CNS diseases related to microglial phagocytosis under pathological conditions are summarized in Table 1.)

**TABLE 1:** Summary of central nervous system diseases related to pathological microglial phagocytosis.

Disease /Animal Model	Subsequence	Type of Pathological Phagocytosis	Target Cell	Molecules /Signaling Pathway	Histological Phenotype	Functional Phenotype	References
Alzheimer's disease: APP/PS1, Aβ, tauP301L	Pathological Mechanism	Excessive	Synapse/Dendritic spines	C1q, mGluR1, TREM, C3, CR3	Aβ plaques and high phosphorylation tau accumulation, synapse loss, glial cell activation	Memory impairment, aphasia and visual impairment	39, 40, 42, 43, 44, 100
Parkinson's Disease: 6‐OHDA, MPTP	Pathological Mechanism	Excessive	Neuronal Cell Body	α‐Syn, FcγR, TREM2, Mrc1, P2Y6, LRRK2, WAVE2	Degeneration and death of dopaminergic neurons in substantia nigra, appearance of Lewy bodies	Tremor, bradykinesia, cognitive impairment	38, 80, 81, 82, 85, 88, 90
Autism Spectrum Disorder: TSC2	Pathological Mechanism	Reduced	Synapse/Dendritic spines	mTOR	Imbalance of glutamatergic signal, imbalance of excitability, abnormal synaptic circuit connections	Abnormal social skills, communication skills impairment, interest's decrease and abnormal behavior patterns	^72^
Schizophrenia: Disc1‐/‐, NRG1‐/‐, Dtnbp1‐/‐	Pathological Mechanism	Excessive	Synapse/Dendritic spines	C3, C4, C4AL	Loss of synapse in prefrontal, temporal, and subcortical structures, injured white matter	Feeling, emotion, behavior, and cognition and the disharmony of mental activities	^50^
Multiple Sclerosis: EAE	Pathogeny	Excessive	Myelin Sheath/Synapse/ Dendritic spines	C1q, C3	Extensive demyelination of white matter, destruction of blood‐brain barrier, proliferation of glial cells, neuroinflammation and synaptic loss	Motor, cognitive and visual deficits	63, 64
Rett Syndrome:MeCP2‐/‐	Pathogeny	Excessive	Synapse/Dendritic spines	Not available	Mature dendritic dendrites of pyramidal neurons contract, the density of dendritic spines decrease	Autism‐like behavior, decreased motor control, irregular breathing, neurodevelopmental disorders	
Ischemic Stroke: MCAO/BCAS Traumatic Brain Injury: TBI	Pathological Mechanism	Excessive	Neuronal Cell Body	C3aR	Massive loss of neurons, extensive axonal injury and demyelination	Hemiplegia, decreased sensory and motor functions, unconsciousness	^97^

## 4 FURTHER PROSPECTS

Microglia, important immune cells of the CNS, are highly activated in many neurological disorders. They proliferate and migrate to the lesion to perform one of their important functions—phagocytosis. Previous studies have suggested that microglia clear necrotic or apoptotic tissue debris and cells, which prevents the release of inflammatory or toxic substances and thereby attenuantes further progression of disease. Most studies focus on the positive effects of microglial phagocytosis; however, in recent years, more and more studies suggest that microglia also phagocyte viable neurons and that this unwarranted phagocytosis is detrimental to tissue regeneration and repair. On the other hand, ASD studies have demonstrated that reduced engulfment of synapses by microglia results in abnormal neural circuit connectivity. The abnormal activation of microglia that leads to excessive or reduced phagocytosis contributes to abnormalities in the structure and function of target cells. It may be a cause of neurological disease or an underlying mechanism that facilitates progression of or exacerbation of diseases. In this review, we term these negative functions of microglia as pathological microglial phagocytosis of the CNS.

Pathological microglial phagocytosis has been implicated in the aberrant activation and expression of "eat‐me" and "don't eat‐me" signals in a variety of CNS disorders. It remains to be investigated whether only one or both of these signaling pathways cause pathological microglial phagocytosis. More associated molecules involved in "eat‐me" or "don't eat‐me" signaling pathway remain to be discovered, such as the newly discovered mannose‐binding lectin (MBL) (lectin) pathway of the complement system, which is suspected to be an important component of the "eat‐me" signaling pathway.^103^ The "don't eat‐me" signaling pathway involves a few molecules, so whether there are other constituent molecules beside SIRPɑ should also be examined. Currently, several studies have focused on the reduced activation of the "don't eat‐me" pathway, so there is a need to investigate whether this pathway is involved in the etiological or pathological process of other diseases in which excessive microglial phagocytosis plays a role.

Previous studies revealed that microglia phagocyte live neurons, live neuronal progenitors, live neutrophils, and live glioma cells, causing death of the engulfed cell,^8^ but it remains elusive whether these microglia are healthy or abnormal. No evidence has revealed whether microglia excessive phagocyte other glial cells yet. Therefore, the root cause of pathological microglial phagocytosis should be further investigated.

The role of excessive microglial phagocytosis in neurological disorders has been studied far more than reduced phagocytosis, and reduced phagocytosis of neurons is virtually non‐existent. Further studies are needed to investigate reduced microglial phagocytosis. Secondary processes surrounding pathological microglial phagocytosis, such as inflammatory responses and the release of ROS, should also be taken into consideration. In terms of the relationship between microglial polarization and phagocytosis, previous studies suggest that microglial anti‐inflammatory polarization promotes phagocytic activity, and activation of the classical pathway of the complement system‐C1q promotes microglial anti‐inflammatory polarization. Is excessive microglial phagocytosis caused by pro‐inflammatory or anti‐inflammatory microglia? A great number of studies suggest that anti‐inflammatory polarization promotes the activation of phagocytic signals in microglia. However, there is a possibility that there is a phagocytic signaling pathway that is activated before anti‐inflammatory polarization, which then causes normal or pathological microglial phagocytosis. Furthermore, there are a few mechanistic studies of signaling pathways and receptors of ASD and Rett syndrome, so they should be further examined. Pathological microglial phagocytosis plays a role in many CNS diseases and serves as a potential therapeutic target. Therefore, further investigation is warranted to uncover the underlying mechanism of pathological microglial phagocytosis to develop potential clinical therapies.

## CONFLICT OF INTEREST

The authors declare no conflict of interest.

## AUTHOR CONTRIBUTIONS

YG designed the review. KW, JL, YZ, YH, DC, and ZS wrote the manuscript. JL drew the figures. AS, YG, KW, and WL critically edited the manuscript.

## Data Availability

This review manuscript has no original data. The authors confirm the absence of shared data.
